# Robot-assisted single-port retroperitoneal partial nephrectomy with a novel purpose-built single-port robotic system with deformable surgical instruments

**DOI:** 10.1007/s00345-024-04827-3

**Published:** 2024-03-13

**Authors:** Chao Zhang, Zheng Wang, Taile Jing, Yong Wei, Fei Guo, Zongqin Zhang, Chengwu Xiao, Yang Wang, Hong Xu, Xiaofeng Wu, Shouyan Tang, Bo Yang, Shuo Wang, Bin Xu, Qingyi Zhu, Dan Xia, Linhui Wang

**Affiliations:** 1 Department Urology, The First Affiliated Hospital of Naval Medical University, Shanghai, 200433 China; 2 Department Urology, The First Affiliated Hospital of Zhejiang University School of Medicine, Hangzhou, 310009 China; 3 Department of Urology, The Second Affiliated Hospital of Nanjing Medical University, Nanjing, 210011 China; 4 Department Urology, The Second Affiliated Hospital of Naval Medical University, Shanghai, 200003 China; 5 Department Urology, Shanghai Ninth People’s Hospital, Shanghai Jiaotong University School of Medicine, Shanghai, 200125 China

**Keywords:** Single-port surgery, Robotic surgery, Partial nephrectomy, Retroperitoneal surgery

## Abstract

**Objective:**

To investigate the safety and feasibility of using a novel purpose-built single-port robotic system (the SHURUI Robotic Surgical System) with deformable surgical instruments to perform retroperitoneal single-port partial nephrectomy.

**Materials and methods:**

A prospective study was conducted to recruit patients with a single renal tumor no more than 4 cm. Robot-assisted single-port partial nephrectomy was performed by using the novel purpose-built single-port robotic system with deformable surgical instruments. Patients’ demographics, tumor characteristics, and perioperative parameters were recorded and analyzed.

**Results:**

Sixteen patients were recruited to the study. The median tumor size was 2.0 cm (IQR: 1.2–2.4 cm). The median R.E.N.A.L score was 6 (IQR: 4–4.5). In 3 cases, pure single-port surgery was carried out, and all the assistance was through the robotic port. Median docking time was 15.5 min (IQR: 14.25–22.25 min). Median operating time was 148.5 min (IQR: 178–238.5 min). Median console time was 107 min (IQR: 92.75–149.75 min). Median warm ischemic time was 26.5 min (IQR: 24.5–30 min). Median blood loss was 17.5 ml (IQR: 10–50 ml).

**Conclusions:**

Retroperitoneal partial nephrectomy can be safely performed with this novel purpose-built single-port robotic system (SHURUI) with deformable surgical instruments. Further studies are needed to fully evaluate the role of this new platform.

## Introduction

The incidence rate of renal cell carcinoma has been increasing during the past decades. It accounts for 5% of all malignancies in men and 3% in women [1, 2]. Because of routine health checkups and convenient imaging examination methods, small renal masses are detected more frequently. For these tumors, partial nephrectomy has been widely accepted [3, 4]. It provides similar oncological outcomes as radical nephrectomy and preserves renal function [5].

Single-port surgery has the characteristics of minimally invasive, rapid post-operative recovery and improved cosmesis [6]. It is the persistent pursuit of urologists. However, due to the “chopsticks effect” and “reverse operation” caused by the parallel arrangement of instruments, the development of laparoscopic surgeries was relatively slow. Robotics provides additional flexibility and may eliminate the problem of “reverse operation” through electronic programming. It has been considered the future of single-port surgeries [7].

Partial nephrectomy can be performed through transperitoneal or retroperitoneal approach. Retroperitoneal surgery, featuring easy renal artery mobilization, accelerated bowel function recovery, and isolation from abdominal organs, is a mature surgical approach [8]. When a single-port robot is applied in a surgery, a relatively large surgical space is needed for deployment of the instruments. The small space of retroperitoneal cavity is challenging. In this study, we introduce a novel purpose-built single-port robotic system with deformable surgical instruments, which is more suitable for retroperitoneal partial nephrectomy.

## Materials and methods

### The purpose-built single-port robotic system with deformable surgical instruments

**Fig. 1:**
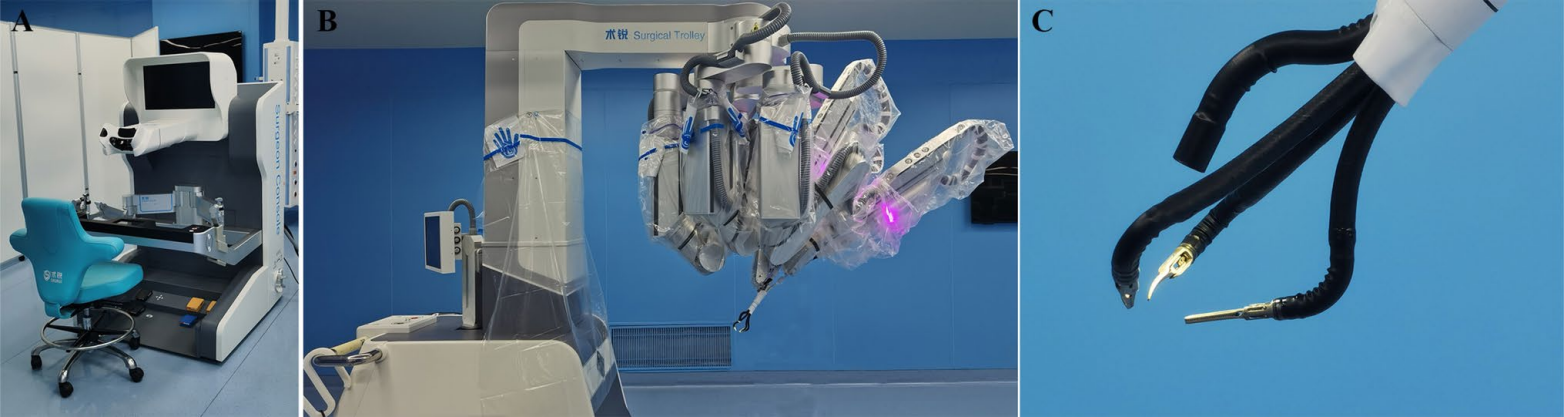
The SHURUI Robotic Surgical System (Model SR-ENS-600; SHURUI Robotics, Beijing, China). **A** Remote console; **B** Operation cart; **C** Deformable surgical instruments

The SHURUI Robotic Surgical System (Model SR-ENS-600; SHURUI Robotics, Beijing, China) consists of a remote-control console with a three-dimensional (3D) high-resolution monitor, a surgical equipment cart with a high-resolution monitor for bedside assistant, a four-arm operation cart, and deformable robotic instruments which are reusable (Fig. 1).

### Partial nephrectomy in clinical application

#### Study design and participants

The multi-center prospective study was conducted from November 2021 to June 2022. Patients with a single renal tumor no more than 4 cm were recruited. Exclusion criteria included lymph node or distant metastasis, body mass index greater than 30, pregnancy, less than 18 years old, presence of infectious diseases, and intolerance to general anesthesia. Written consent forms were obtained from all patients. The study protocol received approval from the Institutional Review Board of each participating center. The respective ethical approval numbers are as follows: the First Affiliated Hospital of Naval Medical University (CHEC2021-143), the First Affiliated Hospital of Zhejiang University School of Medicine (PRO20210241), the Second Affiliated Hospital of Nanjing Medical University (QX-008-LP-01), and Shanghai Ninth People’s Hospital, Shanghai Jiaotong University School of Medicine (SH9H-2021-C53-3).

#### Surgical procedures

**Fig. 2:**
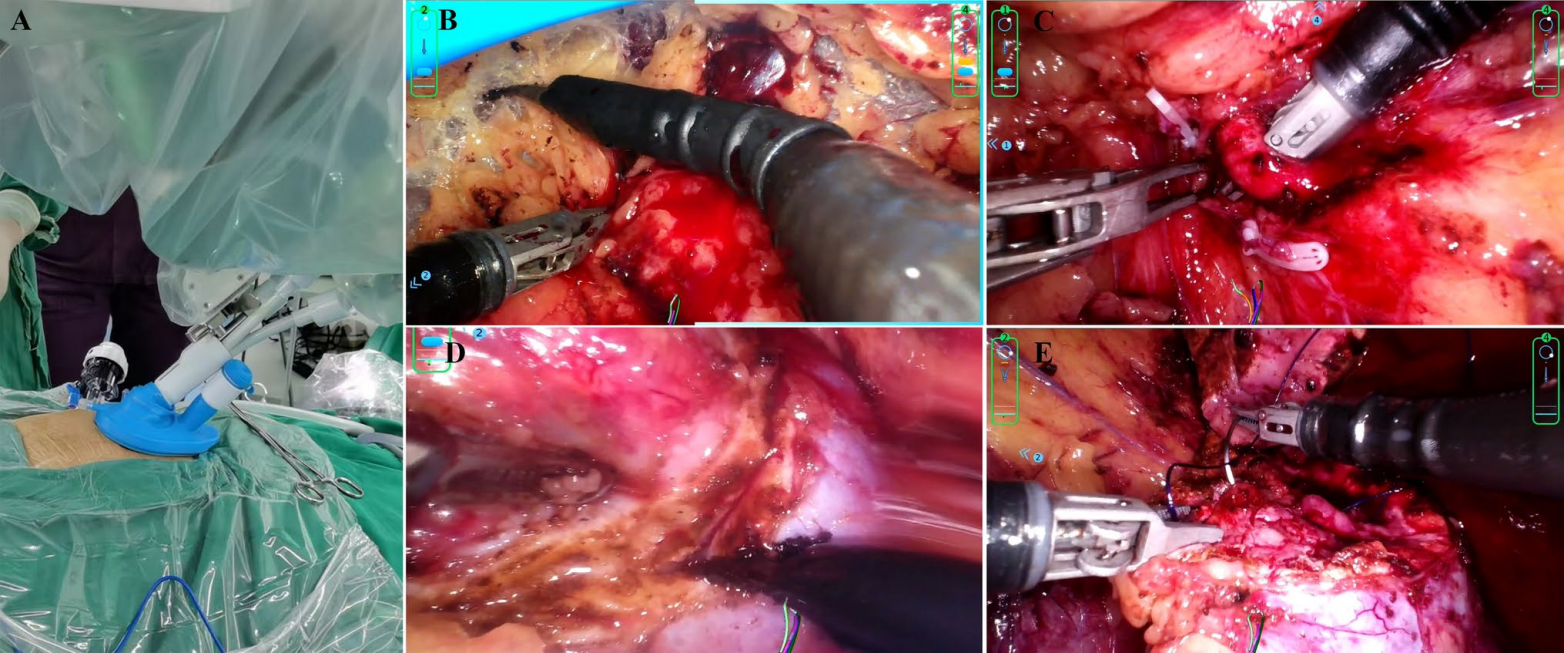
Retroperitoneal partial nephrectomy with the novel purpose-built robotic system with deformable surgical instruments. **A** The single-port trocar in the surgical setting; **B** Mobilization of retroperitoneal fat; **C** Identification of renal artery; **D** Resection of tumor; **E** Renorrhaphy

After general anesthesia, the patient was placed in full lateral decubitus position. One axillary line, a 2.5-cm incision was made 3 cm above the iliac crest. Retroperitoneal space was then established, and robotic port was introduced. A 12-mm assistant port is optional. After docking, 3D high-resolution camera and surgical instruments were installed. The procedure was similar to previous reports [9, 10]. Retroperitoneal fat was removed and Gerota’s fascia was opened. After mobilization of renal artery, pararenal fat was opened to fully expose the tumor. Renal artery was then clamped with bulldog clamps. The tumor was resected, and renorrhaphy was completed running sutures. Bulldog clamps were removed after proper hemostasis. (Fig. 2).

#### Data collection and statistical evaluation

Following information was collected: (1) Patients baseline parameters including age, gender, body mass index (BMI), American Society of Anesthesiologists (ASA) score; (2) Tumor characteristics including side, tumor size, clinical stage, R.E.N.A.L. nephrometry score [11]; (3) Perioperative variables including docking time, operating time, console time, warm ischemic time (WIT), estimated blood loss (EBL), preoperative serum creatinine, serum creatinine at discharge, perioperative complications, and conversions. All complications were evaluated with Clavien–Dindo classification system [12].

Medians and interquartile ranges (IQRs) were used to describe continuous variables. Frequencies and proportions were used to report categorical variables.

## Results

**Fig. 3:**
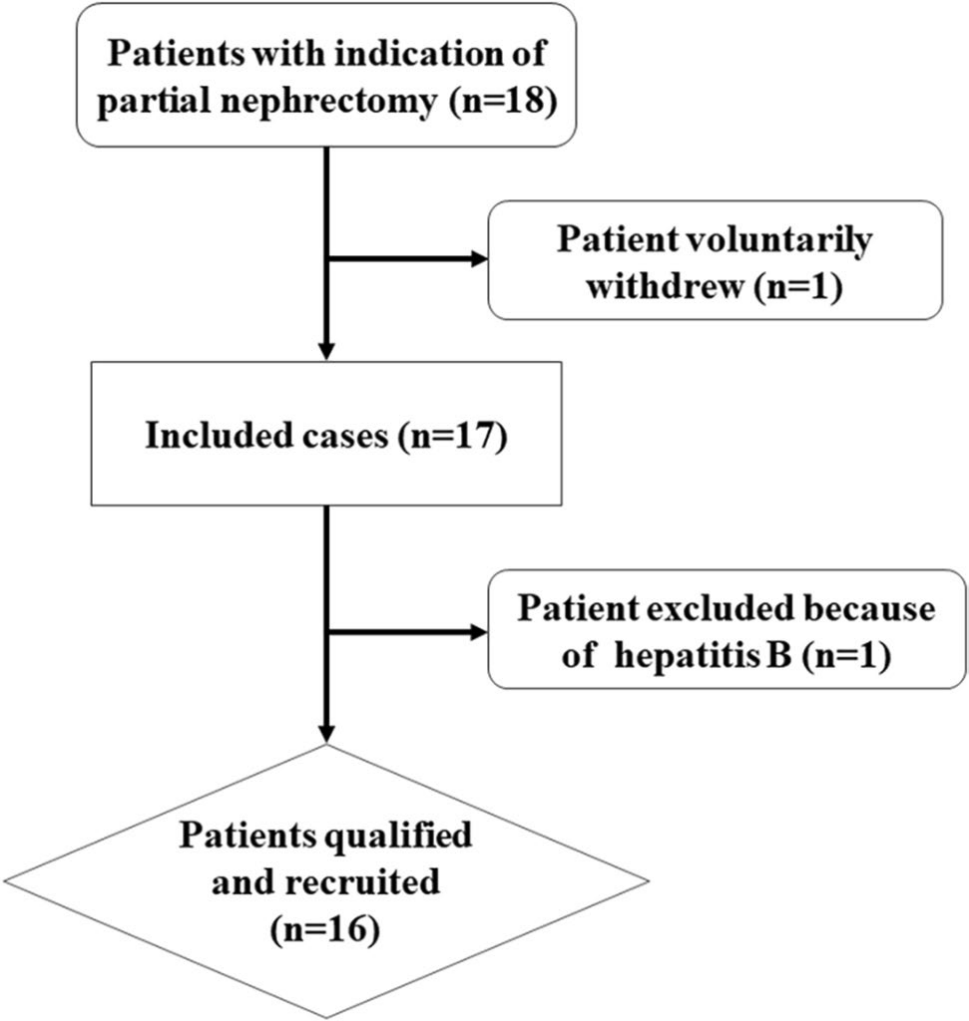
The flow chart of patient recruitment

From November 2021 to June 2022, 18 patients presented with the indication of partial nephrectomy were enrolled with this study. One patient was excluded because of positive hepatitis B antigen and high level of hepatitis B virus DNA. One patient chose not to participate in this clinical trial. Sixteen patients were recruited to this study, and a flow chart is shown in Fig. 3.

The median tumor size was 2.0 cm (IQR: 1.2–2.4 cm). The median R.E.N.A.L score was 6 (IQR: 4–4.5). In 3 cases, pure single port was applied, and all the assistance was through robotic port. The median docking time was 15.5 min (IQR: 14.25–22.25 min). The median operating time was 148.5 min (IQR: 178–238.5 min). The median console time was 107 min (IQR: 92.75–149.75 min). The median warm ischemic time was 26.5 min (IQR: 24.5–30 min). The median blood loss was 17.5 ml (IQR: 10–50 ml). The median preoperative serum creatinine was 68.5 μmol/L (IQR: 58–78.475 μmol/L). The median serum creatinine at discharge was 65.5 μmol/L (IQR: 57.5–89.7 μmol/L). The mean difference in pre- and post-operative creatinine levels was − 0.156 (*p* = 0.952), indicating no significant difference in creatinine levels before and after the operation. All surgeries were successfully performed without major complications or conversions. Patient demographic, tumor characteristics, and perioperative outcomes are shown in detail in Table 1.

**Table 1:** Patient demographic, tumor characteristics, and perioperative outcomes

Variable	Median [IQR] or no. (%)
No. of patients	16
Age (year)	55 [41.5–58]
Male gender	8 (50)
Body mass index	25.6 [23.5–26.8]
ASA score	2 [1, 2]
Right side tumor	9 (56.25)
Tumor size (cm)	2.0 [1.2–2.4]
R.E.N.A.L score	6 [4–6.5]
Docking time (min)	15.5 [14.25–22.25]
Operating time (min)	178 [148.5–238.5]
Console time (min)	107 [92.75–149.75]
Estimated blood loss (ml)	17.5 [10–50]
Warm ischemic time (min)	26.5 [24.5–30]
Major complications (Clavien ≥ 3)	0 (0)
Preoperative serum creatinine (μmol/L)	68.5 [58–78.475]
Serum creatinine at discharge (μmol/L)	65.5 [57.5–89.7]
Positive surgical margin	0 (0)

*ASA* American Society of Anesthesiologists

## Discussion

Compared with traditional laparoscopic surgery, single-port surgery has the characteristics of accelerated recovery and improved cosmetics [6]. When traditional laparoscopic instruments were used, the surgery was very difficult because the instruments were too parallel and the operating angle is narrow. The learning curve was long and relatively simple procedures were performed. Robotics brings a new direction for single-port surgeries. In 2009, Kaouk et al. reported three cases of robot-assisted single-port surgeries with rigid instruments [13]. In 2012, a semirigid robotic operative system was designed to work with da Vinci Si [14]. Thereafter, many studies have achieved satisfying outcomes. However, in these studies, surgeons may only use 2 robotic arms, the operative space is limited, and the flexibility of the instruments decreased significantly [15]. In 2014, Kaouk et al. first reported the application of da Vinci SP platform, and single-port surgery has reached a new height [16]. Yet, the costs and maintenance of da Vinci system remain high, and the SP platform is not yet popularized.

Both transperitoneal and retroperitoneal robotic partial nephrectomies are mature surgical approaches, and each has its advantages and disadvantages. Retroperitoneal surgery provides shorter operating time, less intraoperative blood loss, and faster recovery [17, 18]. Reduced operating time may be related to easy artery identification. Stable pneumoperitoneum pressure may reduce bleeding during tumor resection and renorrhaphy. In addition, retroperitoneal approach is more suitable for patients with abdominal surgery history and abdominal adhesion because it is isolated from abdominal organs [8]. However, reports of retroperitoneal partial nephrectomy with single-port robots are rare. In 2017, Maurice et al. reported 4 cases on male cadavers [19]. In 2020, Kaouk et al. reported the only case of retroperitoneal partial nephrectomy with single-port robot up to present [20]. The patient was a 78-year-old male. Tumor size was 2.5 cm with a nephrometry score of 4. The surgery lasted 242 min, warm ischemic time was 26 min, and estimated blood loss was 25 ml. The operating time was much longer than a routine partial nephrectomy.

The high difficulty explains the small number of retroperitoneal robotic single-port surgeries reported. Retroperitoneal space is an inhospitable environment for deployment of the instruments. Robotic single-port instruments need space to expand and flex. To keep the distance from the operation area, the incision is usually made relatively far away, and operation angle is compromised. In our study, a novel purpose-built single-port robotic system with deformable surgical instruments (SHURUI Surgical System) was used. The surgical workspace of SHURUI robotic surgical system ranges from 7 to 25 cm, which is a significant improvement compared to other purpose-built single-port systems [21]. Up to present, it is the single-port robot with the shortest deployment distance. This feature made it especially suitable for retroperitoneal surgeries. In our series of 16 retroperitoneal cases, all were successful without major complications or conversions. Operating time, warm ischemic time, estimated blood loss were satisfactory.

**Fig. 4:**
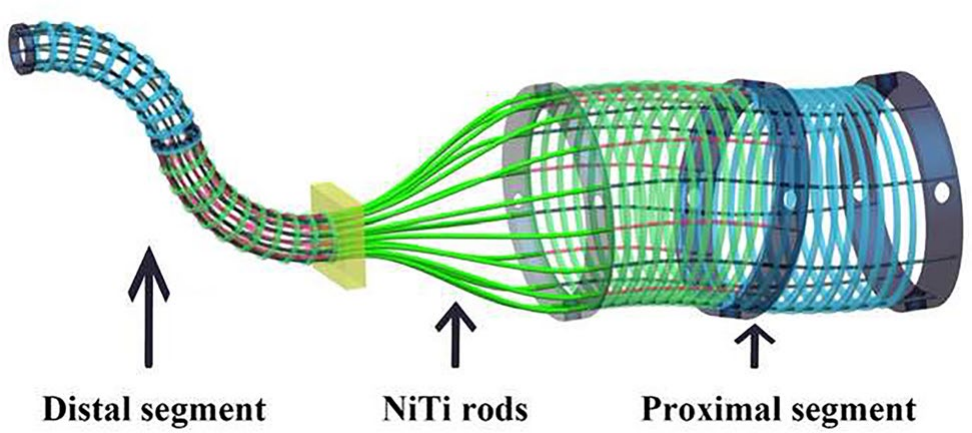
Dual continuum structure of the SHURUI Robotic Surgical System

Unlike cable-driven wrists, a novel structure, dual continuum structure, was applied in SHURUI Robotic Surgical System (Fig. 4). It is composed of the proximal segment, the guide tube and the distal segment. Dozens of nickel–titanium (NiTi) rods run through from the proximal end to the distal end as the structural bones. The bending of the proximal segment will lead to the distal movement to the opposite direction, with a maximum angle of 135 degrees. The dual continuum structure also possesses larger payload capability and greater reliability. Dozens of structural bones bear tension at the same time, enhancing the strength of deformable instruments up to 17.97 Newtons. Additionally, each NiTi Rod influences the surgical tool in a quite small manner. The redundant arrangement of structural bones assures that even crash of a few NiTi rods will not cause the surgery to fail. In separate cohorts, we have also validated the application of this system in radical prostatectomy for prostate cancer, preliminarily confirming its feasibility and safety [22]. Finally, the costs of the SHURUI Robotic Surgical System and its instruments are much lower, which can be as low as laparoscopic surgeries.

There are some limitations of our study and this novel robot. This is a one-arm investigation with a relatively small sample size. Since we were trying to prove the safety and feasibility to perform this surgical approach, BMI was limited to 30, and all tumors were less than 4 cm. Further studies are needed to fully evaluate the role of SHURUI Robotic Surgical System in retroperitoneal partial nephrectomy. Given that this study represents the first clinical application of this novel robotic system, extreme caution was adopted by the surgeons. Tumor excision was carried out carefully, and extra sutures were used during renorrhaphy. This approach was reflected in the relatively prolonged duration of WIT, even for small tumors. The robotic arms of SHURUI Robotic Surgical System occupy a large space. Because of external conflicts, the assistant could only provide limited help. This matter may be improved by flexible laparoscopic instruments which are mature and off the shelf.

## Conclusions

Retroperitoneal partial nephrectomy can be safely performed with this novel purpose-built single-port robotic system with deformable surgical instruments.

## Data Availability

Data supporting the findings of this study are available upon request.

## References

[1] Sung H, Ferlay J, Siegel RL et al (2021) Global Cancer Statistics 2020: GLOBOCAN Estimates of Incidence and Mortality Worldwide for 36 Cancers in 185 Countries. CA Cancer J Clin 71:209–249 33538338 10.3322/caac.21660

[2] Ljungberg B, Campbell SC, Choi HY et al (2011) The epidemiology of renal cell carcinoma. Eur Urol 60:615–621 21741761 10.1016/j.eururo.2011.06.049

[3] Ljungberg B, Albiges L, Abu-Ghanem Y et al (2022) European association of urology guidelines on renal cell carcinoma: the 2022 update. Eur Urol 82:399–410 35346519 10.1016/j.eururo.2022.03.006

[4] Campbell SC, Clark PE, Chang SS et al (2021) Renal mass and localized renal cancer: evaluation, management, and follow-up: AUA guideline: part I. J Urol 206:199–208 34115547 10.1097/JU.0000000000001911

[5] Mukkamala A, He C, Weizer AZ et al (2014) Long-term oncologic outcomes of minimally invasive partial nephrectomy for renal-cell carcinoma. J Endourol 28:649–654 24405274 10.1089/end.2013.0685

[6] Fan X, Lin T, Xu K et al (2012) Laparoendoscopic single-site nephrectomy compared with conventional laparoscopic nephrectomy: a systematic review and meta-analysis of comparative studies. Eur Urol 62:601–612 22704730 10.1016/j.eururo.2012.05.055

[7] Spana G, Rane A, Kaouk JH (2011) Is robotics the future of laparoendoscopic single-site surgery (LESS)? BJU Int 108:1018–1023 21917105 10.1111/j.1464-410X.2011.10513.x

[8] Porreca A, D’Agostino D, Dente D et al (2018) Retroperitoneal approach for robot-assisted partial nephrectomy: technique and early outcomes. Int Braz J Urol 44:63–68 29211396 10.1590/S1677-5538.IBJU.2017.0104 PMC5815533

[9] Ghani KR, Porter J, Menon M et al (2014) Robotic retroperitoneal partial nephrectomy: a step-by-step guide. BJU Int 114:311–313 24571203 10.1111/bju.12709

[10] Harke NN, Darr C, Radtke JP et al (2021) Retroperitoneal versus transperitoneal robotic partial nephrectomy: a multicenter matched-pair analysis. Eur Urol Focus 7:1363–1370 32912841 10.1016/j.euf.2020.08.012

[11] Kutikov A, Smaldone MC, Egleston BL et al (2011) Anatomic features of enhancing renal masses predict malignant and high-grade pathology: a preoperative nomogram using the RENAL nephrometry score. Eur Uorl 60:241–248 10.1016/j.eururo.2011.03.029 PMC3124570 21458155

[12] Dindo D, Demartines N, Clavien PA (2004) Classification of surgical complications: a new proposal with evaluation in a cohort of 6336 patients and results of a survey. Ann Surg 240:205–213 15273542 10.1097/01.sla.0000133083.54934.ae PMC1360123

[13] Kaouk JH, Goel RK, Haber GP et al (2009) Robotic single-port transumbilical surgery in humans: initial report. BJU Int 103:366–369 18778353 10.1111/j.1464-410X.2008.07949.x

[14] Cestari A, Buffi NM, Lista G et al (2012) Feasibility and preliminary clinical outcomes of robotic laparoendoscopic single-site (R-LESS) pyeloplasty using a new single-port platform. Eur Urol 62:175–179 22469392 10.1016/j.eururo.2012.03.041

[15] Bertolo R, Garisto J, Gettman M et al (2018) Novel system for robotic single-port surgery: feasibility and state of the art in urology. Eur Urol Focus 4:669–673 29914841 10.1016/j.euf.2018.06.004

[16] Kaouk JH, Haber GP, Autorino R et al (2014) A novel robotic system for single-port urologic surgery: first clinical investigation. Eur Urol 66:1033–1043 25041850 10.1016/j.eururo.2014.06.039

[17] Kim EH, Larson JA, Potretzke AM et al (2015) Retroperitoneal robot-assisted partial nephrectomy for posterior renal masses is associated with earlier hospital discharge: a single-institution retrospective comparison. J Endourol 29:1137–1142 25816694 10.1089/end.2015.0076

[18] Xia L, Zhang X, Wang X et al (2016) Transperitoneal versus retroperitoneal robot-assisted partial nephrectomy: a systematic review and meta-analysis. Int J Surg 30:109–115 27107660 10.1016/j.ijsu.2016.04.023

[19] Maurice MJ, Ramirez D, Kaouk JH (2017) Robotic laparoendoscopic single-site retroperitioneal renal surgery: initial investigation of a purpose-built single-port surgical system. Eur Urol 71:643–647 27421824 10.1016/j.eururo.2016.06.005

[20] Kaouk J, Aminsharifi A, Sawczyn G et al (2020) Single-port robotic urological surgery using purpose-built single-port surgical system: single-institutional experience with the first 100 cases. Urology 140:77–84 32142725 10.1016/j.urology.2019.11.086

[21] INTUITIVE SURGICAL I. System User Manual Supplement for Abdominal and Pelvic Procedures (U.S. only) https://manuals.intuitivesurgical.com/c/document_library/get_file?uuid=fb25fb1b-ad71-60fd-4bad-50152b102f34&groupId=73750789

[22] Wang Z, Zhang C, Xiao C et al (2023) Initial experience of laparoendoscopic single-site radical prostatectomy with a novel purpose-built robotic system. Asian J Urol 10(4):467–474 38024423 10.1016/j.ajur.2023.08.002 PMC10659971

